# Central autonomic network and early prognosis in patients with disorders of consciousness

**DOI:** 10.1038/s41598-024-51457-1

**Published:** 2024-01-18

**Authors:** Francesco Riganello, Martina Vatrano, Maria Daniela Cortese, Paolo Tonin, Andrea Soddu

**Affiliations:** 1 Reseach in Advanced Neurorehabilitation, S. Anna Institute, 88900 Crotone, Italy; 2 Physics & Astronomy Department and Western Institute for Neuroscience, University of Western Ontario, London, ON Canada

**Keywords:** Diseases of the nervous system, Prognostic markers, Brain injuries, Stroke

## Abstract

The central autonomic network (CAN) plays a crucial role in modulating the autonomic nervous system. Heart rate variability (HRV) is a valuable marker for assessing CAN function in disorders of consciousness (DOC) patients. We used HRV analysis for early prognosis in 58 DOC patients enrolled within ten days of hospitalization. They underwent a five-minute electrocardiogram during baseline and acoustic/visual stimulation. The coma recovery scale-revised (CRS-R) was used to define the patient’s consciousness level and categorize the good/bad outcome at three months. The high-frequency Power Spectrum Density and the standard deviation of normal-to-normal peaks in baseline, the sample entropy during the stimulation, and the time from injury features were used in the support vector machine analysis (SVM) for outcome prediction. The SVM predicted the patients’ outcome with an accuracy of 96% in the training test and 100% in the validation test, underscoring its potential to provide crucial clinical information about prognosis.

## Introduction

Acquired brain injury (ABI) refers to brain damage not related to congenital disorders, developmental disabilities, or progressive neurological conditions^1^. It can result from various causes, such as traumatic brain injury, stroke, infection, hypoxia, or substance abuse, leading to a wide range of cognitive, physical, and behavioral impairments and affecting the level of consciousness.

The term consciousness generally refers to three aspects: the waking state, where individuals can respond to stimuli in an integrated manner; perceptual awareness, denoting a person or animal’s sensory perception; and the intentional state, which encompasses mental states with propositional content, such as beliefs, fears, hopes, expectations, or purposes^2^.

Neurological research identifies two fundamental components of consciousness: wakefulness, which involves spontaneous episodes of eye-opening, and awareness, referring to an individual’s ability to respond in an integrated way to internal and external stimuli^1,3^.

The term disorders of consciousness (DOC) indicates a group of conditions that can emerge following ABI, characterized by altered levels of awareness and responsiveness^4^. These conditions include unresponsive wakefulness syndrome (UWS), also known as vegetative state (VS), minimally conscious state (MCS), and two subcategories of MCS: MCS plus (MCS+) and MCS minus (MCS−)^5,6^, which are generally diagnosed by the coma recovery scale-revised (CRS-R)^1^.

UWS/VS is a condition in which patients exhibit spontaneous eye-opening and sleep–wake cycles, but they lack any signs of awareness or purposeful behavior. In this state, patients are unable to respond to stimuli and demonstrate no evidence of self-awareness or awareness of their environment. MCS is a condition where patients show inconsistent but definite signs of awareness. They may exhibit purposeful behavior, such as following simple commands, verbal or non-verbal communication, or purposeful movement in response to stimuli^1^. MCS patients have a higher level of consciousness than those in UWS/VS but still experience significant limitations in their cognitive and motor functions.

Clinical evidence and neuroimaging research have demonstrated that DOC patients following brain injury can still exhibit preserved modular brain activation and responsiveness, even without the integrated large-network processes typically responsible for consciousness^7,8^. It appears that various network interactions facilitate this remaining responsiveness in DOC^8,9^. In the UWS/VS, activation is limited to lower-level primary sensory cortices without the involvement of higher-order associative cortices, whereas in the MCS, partially preserved activation in higher-order associative cortices has been observed, while the restoration of thalamocortical connectivity has been linked to consciousness recovery^8^. Neuroimaging studies have revealed functional interactions between autonomic nervous structures [i.e., the parasympathetic and sympathetic branches of the Autonomic Nervous System (ANS)] and the neuronal networks involved in higher brain functions, such as attention and conscious processes^10,11^.

**Figure 1:**
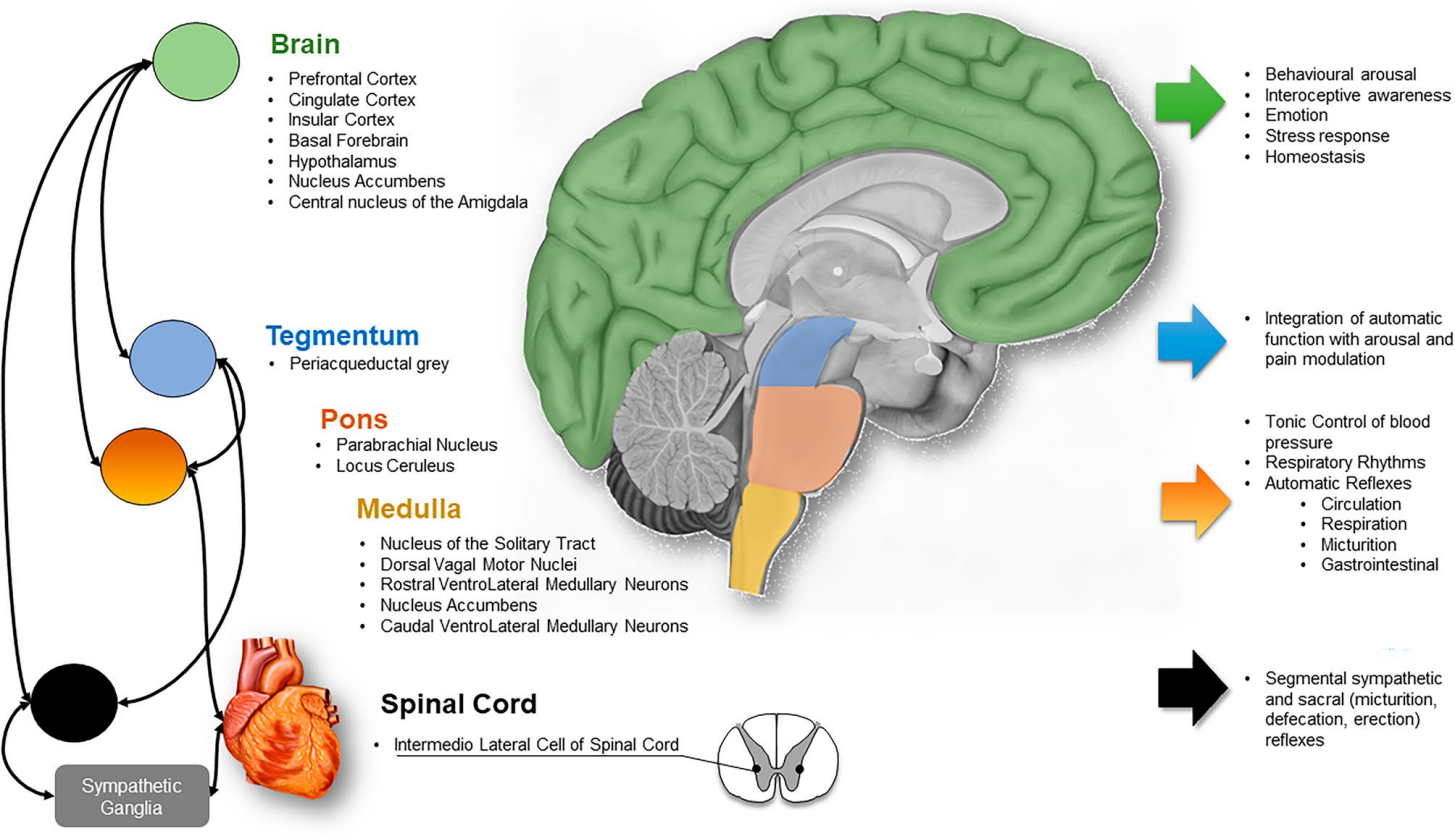
Central autonomic network: schematic representation of the Brain–Heart two-way interaction and relative function of brain areas related to the CAN.

Increasing evidence suggests that ANS function can be non-invasively monitored, and neuroimaging studies have highlighted the reciprocal relationship between the heart and the brain^12,13^. A conceptual model known as the Central Autonomic Network (CAN) has been introduced to depict the two-way interaction between the ANS and central nervous system (CNS), as well as the ongoing modulation of homeostatic processes and allostatic adjustments to internal or external demands^14^ (Fig. 1).

The CAN is functionally organized across several brain regions. The forebrain, which includes the anterior cingulate, nucleus accumbens, insula, ventromedial prefrontal cortex, amygdala, and hypothalamus, engages in bidirectional interactions with both rostral and caudal systems. These forebrain structures are pivotal in integrating visceral and nociceptive inputs, such as thermal and muscular sensations, and in orchestrating autonomic responses to pain and emotional, behavioral, or cognitive stimuli.

Descending to the brainstem, the CAN encompasses the periaqueductal gray, parabrachial nucleus, nucleus of the solitary tract, and the ventrolateral medulla reticular formation, which collectively contribute to autonomic output modulation.

At the spinal level, the CAN operates via neuronal projections that control the segmental reflexive autonomic nervous system. Projections to preganglionic sympathetic and parasympathetic neurons emanate from these brain regions, facilitating the generation of stimulus-specific autonomic response patterns.

This network ensures the body's homeostasis by regulating various functions such as pain modulation, blood pressure, respiratory rhythms, and reflexive actions related to circulation, respiration, micturition, and gastrointestinal processes^15,16^.

Heart rate variability (HRV) has emerged as a valuable and independent indicator of the interaction between the CNS and the ANS in both research and clinical settings. It is characterized by fluctuations in time intervals between consecutive heartbeats and is influenced by the dynamic interplay between sympathetic and parasympathetic activity. The CNS regulates this interplay in response to a wide range of physiological and psychological factors. By analyzing HRV, it is possible to study the intricate relationship between the CNS and ANS, which can provide crucial insights into the overall autonomic system’s health and functioning.

The HRV analysis can be conducted across multiple domains, including time, frequency, and non-linear domains, providing a comprehensive assessment of autonomic function^17,18^. Time-domain (TD) analysis focuses on the evaluation of various statistical measures related to the variability of successive heartbeat intervals, such as the standard deviation of normal-to-normal intervals (SDNN) and root mean square of successive differences (RMSSD), providing insights into the overall HRV and the balance between sympathetic and parasympathetic influences.

Frequency-domain (FD) analysis of HRV involves the examination of power spectral density (PSD), which decomposes HRV into distinct frequency bands, providing insights into the different components of the autonomic nervous system. Typically, three main frequency bands are considered: high-frequency (HF) and low-frequency (LF) band, which ranges from 0.15 to 0.5 Hz and 0.04–0.15 Hz, respectively; and very-low-frequency (VLF) band, which covers frequencies below 0.04 Hz. The HF band is primarily associated with parasympathetic activity, as it reflects the respiratory modulation of heart rate, also known as respiratory sinus arrhythmia^19,20^. On the other hand, the LF band represents a combination of both sympathetic and parasympathetic activity, with the sympathetic component being more dominant^21^. The LF/HF ratio is often used as an index of sympathovagal balance, providing insights into the relative contributions of sympathetic and parasympathetic branches in modulating HRV^21^. Although less understood, the VLF band is thought to be related to long-term regulatory mechanisms, including thermoregulation and the renin–angiotensin–aldosterone system^22,23^.

Non-linear (NL) domain analysis explores the complex and irregular patterns of HRV, which are not easily detected through time or frequency-domain methods. Non-linear metrics, such as entropy measures (e.g., sample entropy and approximate entropy)^24^, fractal measures (e.g., detrended fluctuation analysis)^25^, and Poincaré plots^25^, help to capture the underlying dynamics and irregularities of the autonomic system.

Clinical studies evidenced the diagnostic and prognostic relevance of the HRV analysis in DOC patients after ABI^26–28^. Decreased values of HRV parameters in the different domains were associated with worsened health conditions and mortality in the acute phase^27,29–31^. A recent study evidenced that the HRV analysis provides an accurate outcome prediction at three months on the first day of coma^32^, and their classification by support vector machine (SVM) classification resulted a promising approach^17,33^.

SVM is a powerful supervised machine learning algorithm for classification and regression tasks. Its primary objective is to find the optimal decision boundary that best separates different classes or groups^34^. In the context of prognosis for disorders of consciousness, SVM offers a promising approach to analyzing and interpreting the intricate relationships between HRV parameters and patient outcomes.

Our study aims to predict the outcome of ABI patients with DOC after 3 months of hospitalization in our intensive rehabilitation unit (IRU) by analyzing the HRV at baseline and after auditory and visual stimulation by the SVM approach.

## Results

### Statistical comparisons

No significant difference was found between MCS and UWS/VS groups when compared for age and time from injury at Mann–Whitney test, and no significant difference was found at Chi-square test for sex.

**Figure 2:**
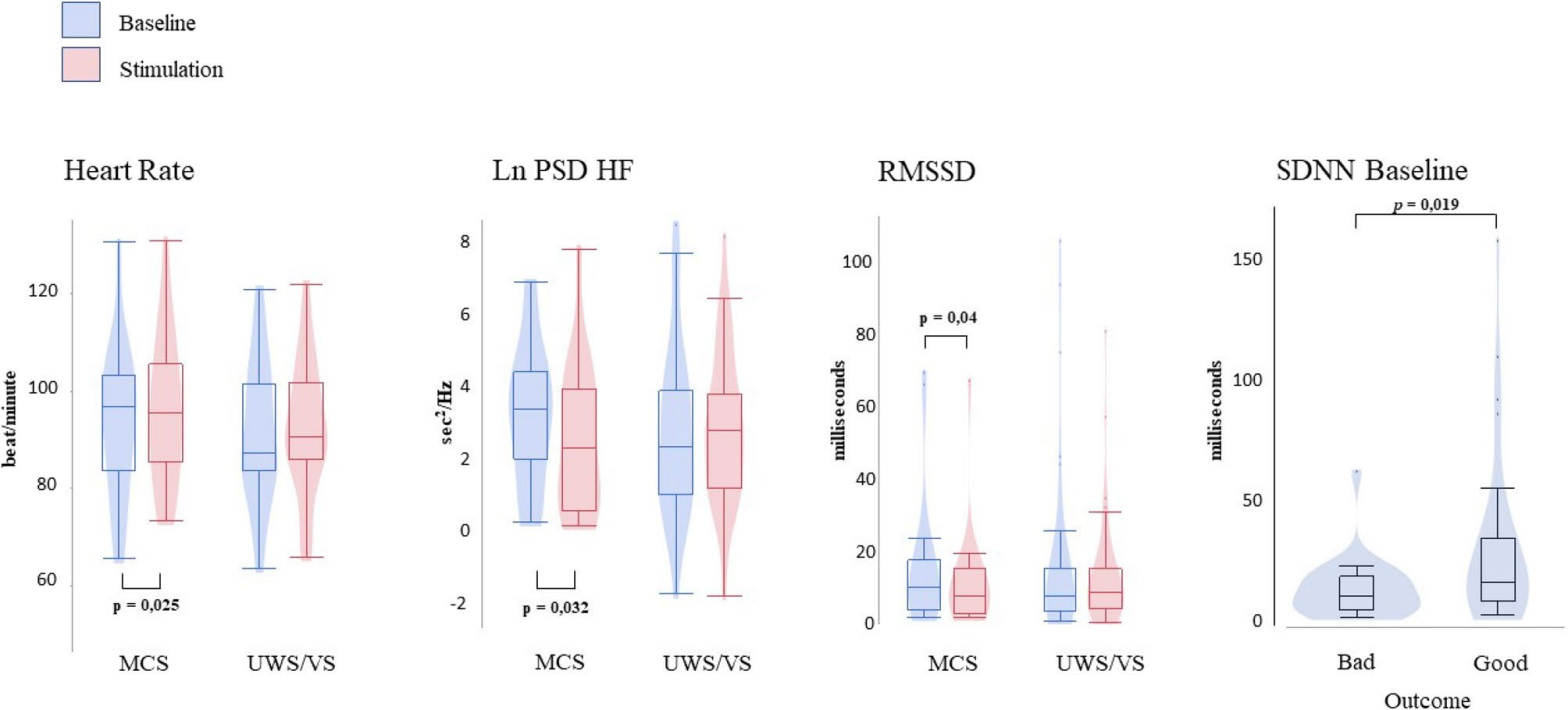
HRV parameters. Boxplot of Heart Rate, natural logarithm of the power spectrum density of the high frequency (Ln PSD HF) and root mean square of successive differences (RMSSD) HRV parameters and comparison between baseline (blue) and stimulation (red). In grey color the standard deviation of normal-to-normal beat (SDNN) of bad and good outcomes.

Significant differences were found between baseline and stimulation in MCS group (N = 19) for HR (Z = − 2.2; p = 0.03; r = 0.36), RMSSD (Z = − 2.0; p = 0.05; r = 0.32), HF (Z = − 2.133; p = 0.03; r = 0.35) (Fig. 2). When comparing HRV parameters between MCS and UWS/VS groups (N = 39), no significant differences were found. When comparing bad and good outcomes, significant difference was found only for SDNN (Z = − 2.1; p = 0.04; r = 0.34) (Fig. 2).

### SVM performance

The input variables used in the SVM model were age, time from injury, aetiology, and HRV parameters (i.e., SDNN, RMSSD, LF, HF, LF/HF ratio, and SampEn) in baseline and stimulation (Table 1). After feature selection, the selected input parameters for predicting bad and good outcomes were HF and SDNN in baseline, SampEn in stimulation, and time from injury.

**Table 1:** HRV input variables used in the SVM model.

Domain	Variable	Definition	Math representation	Math terminology	Psychophysiological meaning
Time	SDNN	The standard deviation of normal-to-normal R–R intervals	$\sqrt{\frac{1}{N-1}\sum {(RR}_{i}-\overline{RR} })$^2^	$\overline{RR }$: mean R–R interval RR_i_: individual R–R intervals N: data length	Overall variability in heart rate over a specific time period
	RMSSD	Root mean square of successive heartbeat interval differences	$\sqrt{\frac{1}{N-1}\sum {(RR}_{i+1}-{RR}_{i}})$^2^	RR_i_: individual R–R intervals N: data length	It reflects the changes in time between successive heartbeats. Higher RMSSD values generally indicate greater parasympathetic nervous system activity
Frequency	PSD LF	Low-frequency band (0.04–0.15 Hz)	Fast Fourier Transform method (Welch’s PSD)		Associated with both sympathetic and parasympathetic activities but often considered a marker for sympathetic activity
	PSD HF	Power spectrum density of High-frequency band (0.15–0.5 Hz)			It is primarily associated with activity in the parasympathetic nervous system
	LF/HF	The ratio between LF and HF power spectrum band	LF/HF		It is used as an indicator of the balance between sympathetic and parasympathetic nervous system activity
Non- linear	SampEn*	Sample entropy	$-{\text{ln}}\frac{{\Phi }^{m}}{{\Phi }^{m+1}}$	**Φ**^**m**^ and **Φ**^**m+1**^ number of matches of length m and m + 1	Assess the unpredictability or irregularity of the intervals between heartbeats. A higher SampEn value indicates greater complexity

*SampEn: Given a sample of length N, the SampEn is defined as the negative natural logarithm of the probability that if two sets of simultaneous data points of length *m* have distance < *r,* then two sets of simultaneous data points of length *m* + *1* also have distance < *r. T*he SampEn parameters *m (i.e., embedding dimension; length of the window of the different vector comparisons)* and *r (i.e., level of tolerance: generally ranging from 0.1 to 0.25, corresponding to the 10–25% of the standard deviation of the series of data onto analysis)* of SampEn were set to 2 and 0.2, respectively.

C and gamma parameters were tested by comparing 100 different models, with C and gamma ranging between 0.5 and 300 and 0.01 and 1, respectively.

**Figure 3:**
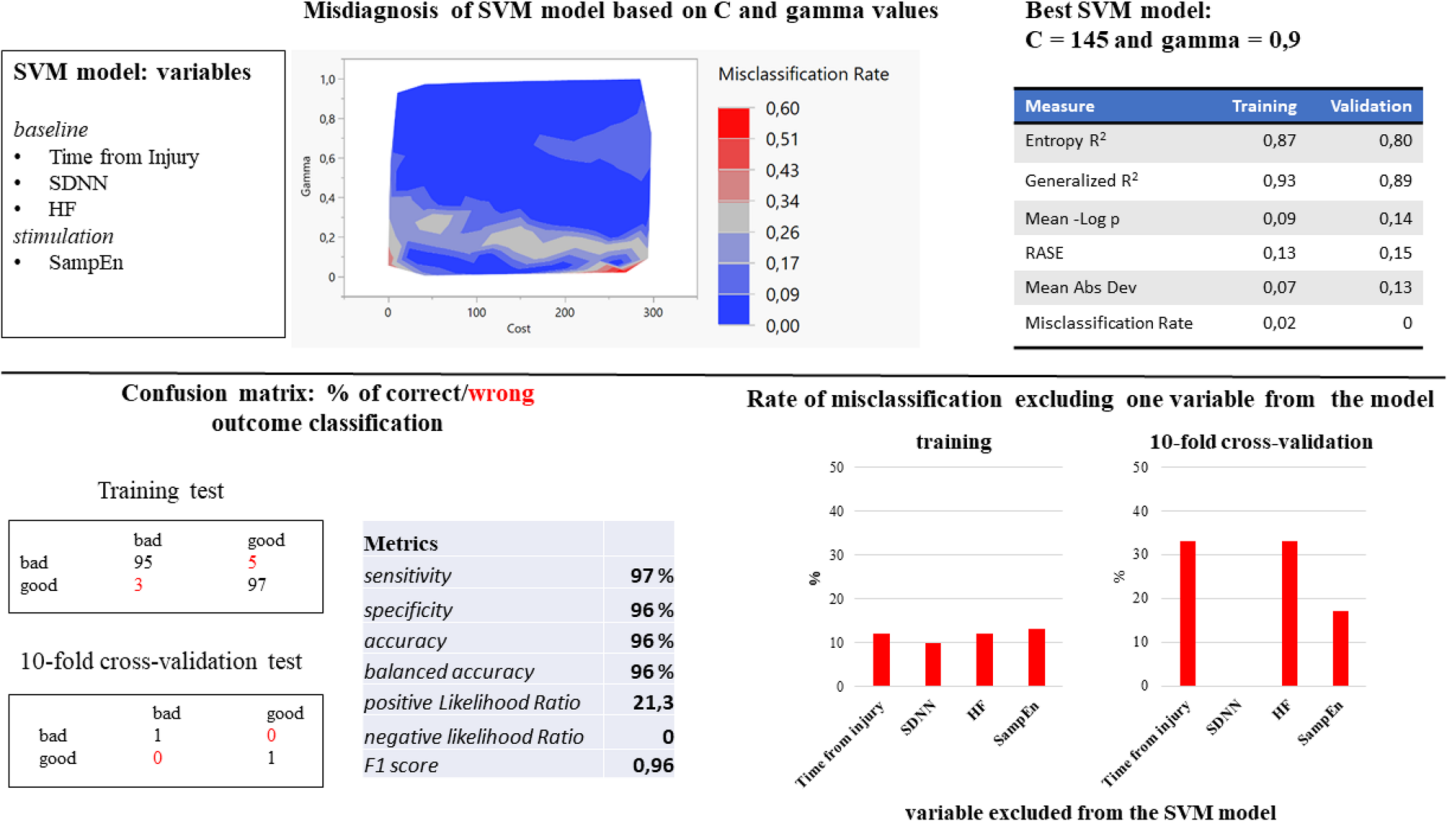
Support Vector Machine (SVM) results. *Line below*: confusion matrix of the SVM model in predicting the patient’s outcome, relative metrics, and misclassification of the model if one of the variables is kept out. Considering the true positive (TP) and false positive (FP) classifications sensitivity (TP/(TP + FN)) and specificity (TN/(TN + FP)) indicate how well the model identifies positive and negatives cases respectively; Accuracy ((TP + TN)/(TP + TN + FP + FN)) measures the proportion of correctly classified cases among the total number of cases; balanced accuracy ((Sensitivity + Specificity)/2) is the average of sensitivity and specificity; positive likelihood ratio (sensitivity/(1 − specificity)) represents how much more likely a positive result is to occur in people with the condition compared to those without it, while the negative likelihood ratio (1 − sensitivity)/specificity represent how much more likely a negative result is to occur in people without the condition compared to those with it; F1 score (2*(precision*sensitivity)/(precision + sensitivity)) measures the model’s accuracy, combining both precision and recall into a single metric, where precision (TP/(TP + FP)) is the ratio between true positives and the sum of true positives and false positives. *Line above*: misclassification rate of 100 SVM models based on cost and gamma values and measure relative to the SVM chosen model. Entropy R^2^ is a goodness-of-fit measure for classification models. It is based on the concept of entropy, which represents the uncertainty in a dataset. Generalized R^2^ measures the proportion of variance in the dependent variable explained by the model. It is an extension of the traditional R-square used for linear regression and can be applied to non-linear models like SVM. Mean-log p (Mean Negative Log Likelihood) measures how well the predicted probabilities from the SVM model match the actual outcomes. RASE (Root Average Squared Error) measures the average squared difference between the predicted and actual values. Mean Absolute Deviation is the average of the absolute differences between the predicted and actual values.

Seventy-one of the models showed misclassification below 10%, and 81% showed misclassification below 15% in the training test, while 67% of the SVM model had no misclassification in the validation test. The best SVM classification was obtained with a C value of 145 and a gamma value of 0.9, with misclassification of 2% in the training test and 0% in the validation test. In the training test, the SVM model had a sensitivity and specificity of 96% and 97%, respectively, and an accuracy of 96% (Fig. 3).

To observe the influence of each variable, the SVM model was computed without the selected variable. Without considering the time from injury, the misdiagnosis in training and validation test was 12% and 33%, respectively; similarly, without considering the HF, the misdiagnosis in training and validation test was 12% and 33%, respectively; without considering the SampEn the misdiagnosis in training and validation test was 13% and 17%, respectively; finally, without considering the SDNN the misdiagnosis in training and validation test was 10% and 0%, respectively (Fig. 3).

## Discussion

Determining the prognosis in DOC patients is a complex task due to the heterogeneous nature of brain injuries, the variability of clinical presentations, and the unpredictable recovery trajectory. However, recent advancements in diagnostic tools and assessment methods have led to improved prognostic accuracy, enabling clinicians to make more informed decisions and tailor treatment plans to the individual needs of patients.

In this context, our study aimed to investigate the potential of using HRV parameters and SVM classification to predict outcomes in patients with UWS/VS and MCS by identifying the relationships between these parameters and the patient’s outcomes in the early stages of their recovery.

We did not find significant differences between MCS and UWS/VS in baseline and stimulation sessions; significant differences were found only for MCS patients during the stimulation sessions, with increased values for HR and decreased values for Ln PSD HF and RMSSD. In fact, during the stimulation, we generally observed an increase in HR in both conditions, which can be associated with an increase in sympathetic activity and, consequently, a reduction of vagal response, as observed in other studies^35,36^. However, in MCS, a better vagal modulation is usually observed, which can also be associated with higher entropy values, indicative of restored functionality at the level of the CAN^11,37,38^. Nevertheless, in our study, the low effect size indicates the low statistical significance of these differences.

Interestingly, the HRV parameters SDNN, Ln PSD of HF, SampEn, and time from injury are effective in the SVM model in predicting the patients’ outcome in the early hospitalization stage. Each HRV parameter belongs to a different HRV domain (i.e., SDNN to the time domain, Ln PSD HF to the frequency domain, and the SampEn to the non-linear domain), and they are not correlated between them.

Clinical and scientific findings indicate that HRV parameters have diagnostic and prognostic significance in DOC independently from the etiology^28,39^. Decreased SDNN and RMSSD were found after TBI in the absence of major DOC^15,30^ as well as, in acute phase, associated with clinical worsening and mortality^27,31,40^. Decreased PSD of the spectral parameters (i.e., HF, LF, and total power) were associated with brain death and increased mortality^29,41,42^. In DOC patients, reduced entropy was found to be an independent predictor of mortality, helping to identify trauma patients at risk of in-hospital death^43–45^.

A study by Lu et al.^46^ on 80 patients with TBI after decompression surgery observed a correlation between HRV parameters and one year of mortality, reporting that SDNN, RMSSD, LF, HF, and PSD total power variables were significantly lower in patients with unfavorable outcomes putting in evidence the impacts the brain–heart axis and cardiac autonomic modulation, in TBI patients, even after decompression surgery.

Another study on TBI patients, based on HRV data collection in the first 24 h of hospitalization in the intensive care unit, evidenced the possibility of using spectral HRV parameters to develop a patient outcome prediction system^47^. Again, the analysis of HRV entropy in 79 DOC patients after cardiac arrest provided accurate outcome prediction at three months on the first day of coma^32^, where higher entropy levels correlated to a better outcome.

All these findings evidence the utility of HRV analysis in developing models to predict patient outcomes after ABI.

HRV metrics, encompassing time, frequency, and nonlinear domains, provide crucial insights into the functionality of the ANS. These measures shed light on the CAN, which integrates higher brain functions with autonomic processes, and HRV is posited as a key indicator of this integration^12,13,16,48,49^. HRV metrics also capture the dynamics of physiological factors that influence cardiac rhythm and its adaptive response to varying internal and external stimuli^35,36,38^. This modulation is a function of the heart and peripheral mechanisms and central structures like the insula, a key structure within the CAN that plays a pivotal role in modulating HRV, as it integrates autonomic, affective, and cognitive information^50–52^. The HRV analysis provides information on the brain–heart two-way interaction and the continuous modulation of homeostatic processes and allostatic adaptation to internal or external requirements^12,14^.

In this frame, the HRV analysis could play a central role in clinical applications, particularly in assessing the prognosis of patients with DOC. Reflecting the integrative function of the CAN, the HRV metrics offer valuable prognostic insights in these patients.

In our model, the time from injury was also critical in providing better model accuracy. The importance of this parameter was suggested in other studies^53–56^. In a multicenter study, a short time from injury was reported as significant predictor in predicting a better outcome, jointly to younger age, higher CRS-R total score, and presence of EEG reactivity to eye opening^57^.

The significant effect of the time elapsed since injury in predicting the outcome could be related to the fact that patients with more severe brain injuries are typically admitted to rehabilitation settings later^53,58,59^.

Our findings suggest that time from injury, jointly with reduced variability of the HR (SDNN), vagal tone (Ln PSD HF), and reduced HR complexity (SampEn) during the stimulation, are factors that could help in predicting the patient’s outcome.

The results confirm the importance of an early assessment of brain–heart interaction in patients with DOC. The HRV analysis represents a practical method that can provide crucial information used in the context of a multidisciplinary approach^1^ comprehensive of behavioral and instrumental (i.e., EEG, evoked potential, medical imaging techniques such as magnetic resonance imaging and others) assessment.

In our study, the multidimensional approach, represented by the HRV parameters from different domains (time, frequency, and non-linear), comprehensively describes the patients’ condition brain–heart interaction. More, the controlled environmental parameters, such as noise, temperature, humidity, and luminosity, represent a significant strength. By maintaining a stable environment condition, we minimized the potential impact of external factors on the HRV measurements, ensuring more accurate and reliable data. This control contributes to the validity of our findings, as it reduces the likelihood that variations in HRV parameters are due to extraneous influences. Consequently, the results provide a clearer picture of the relationship between HRV parameters and patient outcomes.

In our study, the use of SVM classification demonstrates the potential of machine learning algorithms in aiding clinical decision-making and predicting outcomes in patients with UWS/VS and MCS. More, applying ten-fold cross-validation enhances the reliability and generalizability of the SVM model. The ten-fold cross-validation ensures the robustness of the model and the higher likelihood of success when applied to new patient cases.

However, some limitations are present. We selected specific HRV parameters that are commonly analyzed in the study of patients with disorders of consciousness^11^, such as the SDNN, PSD HF and SampEn. Nonetheless, other analytical techniques, including the Poincaré plot^60,61^ and the detrended fluctuation analysis^62,63^, or multiscale entropy^45^, which also describe heart rate dynamics in depth, could offer additional insights into predicting changes in consciousness levels in this patient population.

The study’s findings may be limited in generalizability due to the small sample size of patients. Future research with larger samples is necessary to validate the results. The heterogeneous nature of brain injuries and the variability of clinical presentations may also limit the applicability of the results to all patients with disorders of consciousness. Another limitation is that our study focuses on the early stages of recovery, and it would be beneficial to assess the patient’s progress over time and investigate the longitudinal relationship between HRV parameters and patient outcomes, allowing a more nuanced understanding of the factors influencing prognosis in DOC patients.

Despite these limitations, our study provides valuable insights into the potential of HRV analysis and SVM classification in predicting outcomes for patients with DOC. Further research with larger sample sizes, diverse patient populations, and longitudinal assessments will help refine these prediction models and contribute to developing more effective, patient-centered treatment strategies.

## Methods

### Subjects

The study enrolled 58 patients discharged from the intensive care unit after the acquired brain injury and diagnosed with DOC of different etiology within the first 10 days of hospitalization at the IRU of S. Anna Institute. Thirty-nine were UWS/VS (16 females, age 51 ± 8, 2 Anoxic (ANOX), 7 hemorrhagic (HEM), 1 ischemic (ISC), 2 oncologic (ONC), 4 traumatic brain injury (TBI); 23 males, 5 ANOX, 7 HEM, 2 ISC, 9 TBI), and nineteen were MCS (10 females, age 53 ± 17, 7 HEM, 1 ISC, 1 ONC, 1 TBI; 9 males, age 38 ± 15, 3 HEM, 1 ISC, 5 TBI), with an intercourse time from injury and hospitalization of 51 ± 28 days for UWS/VS, and 57 ± 39 days for MCS (Table 2). Inclusion criteria were age more than 16, no administration of neuromuscular blockers or sedation within 24 h of enrolment, diagnosis of UWS or MCS, based on behavioral assessments using the CRS-R; exclusion criteria were clinical instability, sepsis, COVID-19 infection, and previous neurological or psychiatric disorders.

**Table 2:** Demographic information.

MCS									UWS/VS								
Patient	Sex	Age	Aetiology	Outcome at 3 months	Outcome good/bad	CRS-R within 10 days	CRS-R at 3 months	Time from injury (days)*	Patient	Sex	Age	Aetiology	Outcome at 3 months	Outcome good/bad	CRS-R within 10 days	CRS-R at 3 months	Time from injury (days)*
1	F	65	HEM	MCS	Good	9	12	54	20	F	39	ANOX	UWS/VS	Bad	6	6	28
2	F	71	HEM	MCS	Good	11	12	36	21	F	36	ANOX	MCS	Good	5	11	55
3	F	68	HEM	MCS	Good	13	13	27	22	F	76	HEM	DEAD	Bad	3	0	26
4	F	47	HEM	MCS	Good	13	13	60	23	F	64	HEM	UWS/VS	Bad	5	5	31
5	F	39	HEM	MCS	Good	13	14	54	24	F	71	HEM	UWS/VS	Bad	3	6	68
6	F	51	HEM	EMC	Good	13	17	71	25	F	71	HEM	UWS/VS	Bad	6	6	76
7	F	22	HEM	EMC	Good	11	21	51	26	F	56	HEM	UWS/VS	Bad	6	6	54
8	F	63	ISC	MCS	Good	14	14	23	27	F	39	HEM	UWS/VS	Bad	4	7	45
9	F	71	ONC	DEAD	Bad	9	0	182	28	F	53	HEM	MCS	Good	7	11	33
10	F	32	TBI	MCS	Good	14	14	112	29	F	73	ISC	EMC	Good	3	19	38
11	M	56	HEM	MCS	Good	15	16	62	30	F	66	ONC	UWS/VS	Bad	1	4	126
12	M	54	HEM	EMC	Good	15	17	45	31	F	51	ONC	EMC	Good	3	18	55
13	M	22	HEM	EMC	Good	11	20	30	32	F	23	TBI	DEAD	Bad	3	5	32
14	M	58	ISC	MCS	Bad	13	10	43	33	F	54	TBI	MCS	Good	5	9	37
15	M	27	TBI	MCS	Good	10	10	17	34	F	22	TBI	MCS	Good	6	9	93
16	M	39	TBI	MCS	Good	9	12	49	35	F	29	TBI	MCS	Good	8	11	39
17	M	24	TBI	MCS	Good	9	15	97	36	M	69	ANOX	UWS/VS	Bad	1	4	26
18	M	21	TBI	EMC	Good	11	17	47	37	M	62	ANOX	UWS/VS	Bad	5	5	49
19	M	45	TBI	EMC	Good	15	19	27	38	M	56	ANOX	UWS/VS	Bad	5	6	40
									39	M	28	ANOX	MCS	Good	4	14	63
									40	M	33	ANOX	MCS	Good	7	16	33
									41	M	57	HEM	UWS/VS	Bad	2	5	51
									42	M	53	HEM	UWS/VS	Bad	4	5	27
									43	M	51	HEM	UWS/VS	Bad	6	6	33
									44	M	56	HEM	UWS/VS	Bad	4	7	122
									45	M	18	HEM	UWS/VS	Bad	7	7	110
									46	M	63	HEM	UWS/VS	Bad	3	8	60
									47	M	74	HEM	MCS	Good	3	12	22
									48	M	54	ISC	UWS/VS	Bad	2	2	48
									49	M	46	ISC	MCS	Good	4	9	21
									50	M	18	TBI	UWS/VS	Bad	4	4	48
									51	M	33	TBI	UWS/VS	Bad	3	6	122
									52	M	39	TBI	UWS/VS	Bad	5	6	50
									53	M	30	TBI	UWS/VS	Bad	7	6	39
									54	M	32	TBI	MCS	Good	5	12	35
									55	M	26	TBI	MCS	Good	6	14	51
									56	M	13	TBI	MCS	Good	8	16	43
									57	M	14	TBI	MCS	Good	4	18	51
									58	M	32	TBI	EMC	Good	8	22	22

*UWS/VS* unresponsive wakefulness syndrome/vegetative state, *MCS* minimally conscious state, *EMC* emersion, *HEM* hemorrhagic, *TBI* traumatic brain Injury, *ANOX* anoxic, *ISC* ischemic, *ONC* oncologic, *CRS-R* coma recovery scale-revised.

*Time from injury is the intercourse time from acute event and hospitalization at S. Anna Institute.

### Procedure

Within 10 days of hospitalization, an expert neuropsychologist assessed the patient’s consciousness level with the CRS-R, seated in the wheelchair or semi-reclined on the bed, in a room without transient noise and with constant temperature (24–25 °C), luminosity (300 lx), and humidity (40%). Before the assessment, 5 min of baseline and 5 min of stimulation were recorded by electrocardiogram (ECG). According to the CRS-R procedure, the patients were stimulated with auditory and visual stimuli.

The CRS-R assesses brainstem, cortical, and sub-cortical functioning and consists of 23 items divided into six subscales (i.e., auditory, visual, motor, oromotor/verbal, communication, and arousal subscales), hierarchically arranged. Its scoring is based on the presence/absence of specific behavioral reactions to standardized sensory stimuli, with the lowest items of the scale representing a reflexive activity and the higher items a cognitively mediated response^64^.

The three lead ECGs were recorded by the Nexus 10 using adhesive electrodes positioned on the left clavicle (white), right clavicle (black), and left lower chest (red). Since a sample frequency rate of the signal acquisition below 250 Hz can affect the HRV analysis, the signal was acquired with a sample rate of 256 Hz^65^.

The study was conducted in accordance with the Declaration of Helsinki and was aproved by the Institutional Review Board of "Istituto S. Anna", with the guidance of the Scientific Director (n.ro 5/fr, October 21, 2022). Written informed consent was obtained by the patient's legal representatives.

### Data analysis

ECG-acquired data during the 5 min of baseline and stimulation were analyzed by Kubios advanced software for HRV analysis (v 3.1/Kuopio, Finland), and parameters were extracted in time, frequency, and non-linear domains. The acquired signal was checked for noise, and then the R peaks were detected by applying the Kubios QRS detection algorithm based on the Pan–Tompkins algorithm^66^. The 4 Hz cubic spline interpolation was applied to extract the R-peaks correctly (i.e., to eliminate artifacts and ectopic beats from the data). Further, the signal was ulteriorly visually checked for the R peak corrected acquisition and manually adjusted if necessary. Since the complex nature of the biological systems, the heart rate signal is non-stationary and frequently contains either slow trends or very slow frequency oscillations. As the HRV parameters may be influenced by the non-stationary nature of the signal, a quadratic polynomial model was employed to detrend the RR series, thereby minimizing the impact of lower frequencies on the PSD results. The heart rate (HR), the RMSSD, and the SDNN were calculated in the TD. The natural logarithm of the HRV PSD of HF (0.15–0.5 Hz) (LnHF), LF (0.04–0.15 Hz) (LnLF), and LF/HF ratio were computed in the FD domain using the Fast Fourier Transform method (Welch’s PSD; windows width: 150 s.). The transformation of the spectral power in their natural logarithm was applied because the measures showed a skewed distribution (i.e., skewness > 4.2). Finally, the sample entropy (SampEn) was calculated in the NL domain.

### Statistical analysis

MCS and UWS/VS groups were compared for HRV parameters by the two-tailed Mann–Whitney test, and the baseline and stimulation conditions were compared for each group by the two-tailed Wilcoxon test.

HRV parameters of MCS and UWS/VS groups were compared based on the Mann–Whitney exact test, while the baseline and stimulation conditions were compared for each group by the Wilcoxon test. The effect size was calculated as the absolute value of Z/√(N) for the Mann–Whitney test and the absolute value of Z/√(2*N) for the Wilcoxon test, where Z is the Z-statistic of the statistical test, and N is the total number of subjects. The effect size results were considered as follows: r < 0.1, not significant; 0.1 ≤ r < 0.3, low; 0.3 ≤ r < 0.5, medium; r > 0.5, high.

### Outcome

Due to the limited sample size, it was chosen to group all MCS patients (i.e., including MCS−, MCS+, and emerged from MCS) together for SVM modeling to achieve a robust binary classification. This dichotomization (i.e., good versus bad outcome) was necessary to ensure the statistical reliability and interpretability of the SVM outcomes. The level of consciousness was assessed three months post-hospitalization in the IRU of S.Anna Institute, using the CRS-R. A 'good outcome' was defined as any UWS/VS patient who progressed to any MCS state, and any MCS patient who either demonstrated an increase in or maintained their CRS-R total score.

### Support vector machine

By JMP software (version 16, SAS Institute, Cary, NC), the SVM technique was used to classify bad and good outcomes. SVMs work by finding the best boundary that separates different groups in the data while maximizing the distance between them. This makes SVM particularly useful for handling complex data sets, as they can strike a balance between fitting the data too closely (overfitting) and generalizing the model for better performance on new, unseen data.

To enhance the SVM capabilities, a method called the Radial Basis Function (RBF) kernel was used. The RBF kernel helps transform the input data into a more manageable format, making it easier to separate data points that might otherwise be difficult to distinguish. Using the RBF kernel with the SVM makes it possible to efficiently handle complex relationships between the data points and achieve better classification results. In other terms, the combination of SVM and RBF kernel allows us to create a more flexible and accurate model that can adapt to various patterns and trends in the data.

In the SVM model, the cost (C) and RBF gamma parameters play a crucial role in determining the model’s performance. The C parameter controls the balance between maximizing the margin (i.e., the distance between the decision boundary and the nearest data points) and minimizing the classification error, while the RBF gamma parameter determines the shape of the decision boundary by controlling the flexibility and smoothness of the curve. Tuning both C and gamma values is crucial to achieve an optimal balance between model complexity and generalization performance, ensuring the SVM model performs well on unseen data.

To ensure the robustness and reliability of the SVM model, a technique called ten-fold cross-validation was employed for training and validation purposes. Cross-validation is a widely used method to assess the performance of a model by dividing the data into multiple subsets and training the model on each subset while validating it against the remaining data. Ten-fold cross-validation is a robust method for assessing the performance of a machine-learning model. In this procedure, the original dataset is randomly partitioned into ten equal-sized subsets or “folds”. Each of these subsets comprises approximately 10% of the data. The model is then trained and validated ten times, each time using a different subset as the validation set, and the remaining 90% of the data (the other nine subsets) as the training set. In this method, each individual data point is included in the validation set one time only and contributes to the training set in nine out of the ten iterations. After these iterations, the model’s performance is computed by taking the average across all ten rounds of validation^67^.

By using ten-fold cross-validation, we can minimize the risk of overfitting and ensure that our model generalizes well to new, unseen data. This method provides a more accurate estimate of the model’s performance, as it reduces the impact of any single training set on the overall performance metrics. Furthermore, it allows us to evaluate the consistency of our model’s performance across different training and validation sets, which is crucial for assessing its reliability and robustness in real-world applications.

## Data Availability

The datasets generated and/or analyzed during the current study are not publicly available due privacy/ethical restrictions but are available from the corresponding author on reasonable request.
